# Channel-assisted cervical key hole technology combined with ultrasonic bone osteotome versus posterior percutaneous endoscopic cervical foraminotomy: a clinical retrospective study

**DOI:** 10.1007/s00264-023-05991-8

**Published:** 2023-10-02

**Authors:** Xiao Sun, Chuanen Wang, Qingquan Kong, Bin Zhang, Pin Feng, Junlin Liu, Yuan Hu, Junsong Ma, Junwei Xiang

**Affiliations:** 1 Department of Orthopedics, West China Hospital, Sichuan University, Chengdu, Sichuan China; 2 Department of Pain, Hospital of Chengdu Office of People’s Government of Tibetan Autonomous Region, Chengdu, Sichuan China; 3 Minimally Invasive Area of the Spine, The Affiliated Hospital of Chengdu Sport University, Chengdu, Sichuan China; 4 Department of Orthopedics Surgery, Hospital of Chengdu Office of People’s Government of Tibetan Autonomous Region, Chengdu, Sichuan China

**Keywords:** Key hole, Cervical spondylotic radiculopathy, Ultrasonic bone osteotome, Clinical effect

## Abstract

**Purpose:**

The search for more effective and safe treatment methods for cervical spondylotic radiculopathy (CSR) has led to the rapid development and increasing popularity of minimally invasive posterior cervical foraminotomy (MI-PCF). This study aims to compare two important approaches for MI-PCF surgery: the channel-assisted cervical key hole technology combined with ultrasonic bone osteotome (CKH-UBO) and posterior percutaneous endoscopic cervical foraminotomy (PPECF).

**Methods:**

Data from patients treated with single-level CKH-UBO (*n* = 35) or PPECF (*n* = 40) were analyzed. Clinical outcomes, including visual analogue scale (VAS) scores for neck and arm pain, Neck Disability Index (NDI), and modified Macnab criteria, were assessed preoperatively, as well as at three days, three months, and one year postoperatively.

**Results:**

The percentages of patients with excellent and good outcomes were 97.14% and 92.5%, respectively. The average surgical time in the CKH-UBO group was significantly shorter than in the PPECF group (*p* < 0.001), while the average incision length in the PPECF group was significantly smaller than in the CKH-UBO group. There were no significant differences between the two groups in terms of blood loss, hospital stay, and clinical outcomes at three days, three months, and 12 months postoperatively.

**Conclusion:**

CKH-UBO can achieve the same surgical outcomes as PPECF for the treatment of CSR. However, CKH-UBO saves more time but requires patients to undergo larger incisions.

## Introduction

The gold standard for conservative treatment-resistant cervical spondylotic radiculopathy (CSR) is anterior cervical discectomy and fusion (ACDF). However, for single-segment CSR, non-fusion decompression surgery is commonly used to avoid adjacent segment degeneration and reduce the inefficiency associated with fusion [1–5]. Non-fusion decompression techniques can be performed through anterior or posterior approaches, each with its own advantages [6, 7]. Among these, the posterior approach, specifically minimally invasive posterior cervical foraminotomy (MIS-PCF), has been shown to achieve more adequate decompression of the nerve root canal [3, 8], making it a popular choice for its effectiveness [9, 10].

Key hole technology is an important facet of MIS-PCF, allowing limited yet efficient posterior laminar window creation, which is crucial for ensuring high efficiency [11, 12]. Various auxiliary keyhole techniques have been developed to enhance the exposure. These include endoscope-assisted procedures [13] as well as the use of larger channels or microscopes [14]. In terms of decompression methods, ultrasonic bone osteotome (UBO) has emerged as a new technique that allows faster bone cutting compared to traditional methods, without causing damage to surrounding soft tissues due to its ultrasonic frequency setting [15]. Importantly, with the introduction of UBO, the combination of a large channel approach and a microscope is no longer mandatory. A surgical approach using a large channel combined with UBO has been developed, potentially significantly improving the efficiency of MIS-PCF surgery [16].

Our clinical team currently focuses on two types of MIS-PCF procedures. The first is PPECF, which we have been performing for many years. The second is CKH-UBO, which we gradually started incorporating into our practice within the past five years and is now commonly used. However, we currently lack a definitive consensus on which approach holds an advantage in terms of safety, efficiency, and short-term and long-term clinical outcomes. To the best of our knowledge, there are no studies comparing the efficacy of these two surgical techniques conducted by other centers. This article aims to conduct a retrospective comparative study of these two surgical approaches.

## Methods

### Patients

We enrolled consecutive cases of cervical spondylotic radiculopathy (CSR) diagnosed and treated with either CKH-UBO or PPECF in our clinical research team from March 2020 to January 2022. Both groups of patients strictly adhered to the inclusion and exclusion criteria. The inclusion criteria were as follows: (1) a confirmed diagnosis of CSR; (2) ineffective conservative treatment for at least three months; (3) unilateral radicular pain, such as pain, numbness, weakness, with or without neck pain, and with consistent findings on magnetic resonance imaging (MRI) and computed tomography (CT) showing unilateral single segment foraminal stenosis, possibly accompanied by a focal protrusion of the same level of intervertebral disc on the extreme lateral side. Exclusion criteria included the presence of other cervical spine pathologies such as severe cervical curvature changes, cervical instability or subluxation, severe myelopathic cervical spondylosis, cervical infection, tumor, or fracture, and neck or upper limb pain and numbness caused by other reasons. The choice between CKH-UBO or PPECF was based on the mutual decision of the surgeons and the patients.

Finally, a total of 76 patients were included in our study within the specified research period, with 36 undergoing CKH-UBO and 40 undergoing PPECF. One CKH-UBO patient was excluded from the study at the six month postoperative mark due to injury from a car accident.

### Surgical techniques

**Fig. 1:**
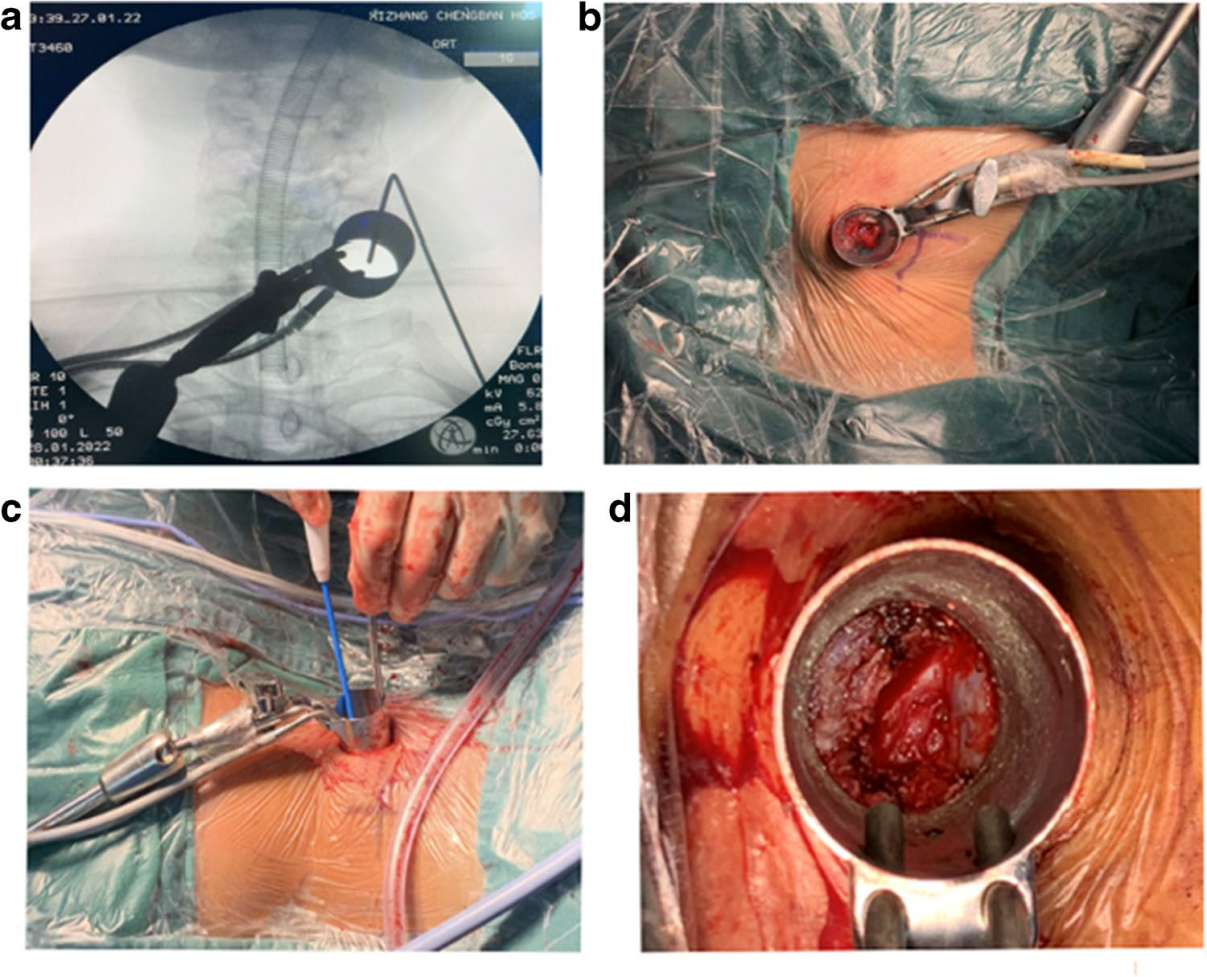
Intraoperative perspectives of the CKH-UBO procedure: **a** C-arm positioning; **b** overview of the large channel; **c** clearing the field of vision and achieving hemostasis through the large channel; **d** view within the large channel

All patients underwent general anaesthesia and were placed in a prone position on a surgical table with support to the head and cervical spine using Macintosh’s headrest, with slight flexion of the neck. The intervertebral space of the lesion was determined through fluoroscopy. The incision site was selected approximately 2 cm lateral to the midline on the affected side. From this point, the surgical steps and approaches differ for each, as described below:

- PPECF: After determining the desired height using C-arm fluoroscopy, an approximately 8-mm lateral skin incision parallel to the joint space was made, followed by dissection of the fascia. A gradual soft tissue working channel was established along the incision. Once the C-arm fluoroscopy confirmed satisfactory positioning, a working sleeve was further inserted using a retractor. When the working sleeve was seen to be located within the joint space and just medial to the protrusion of the articular process, it was connected to the endoscope with a saline irrigation system. Under endoscopic visualization, the surrounding soft tissues around the facet joints were cleared using a bipolar radiofrequency electrode to expose the V-point located between the upper and lower laminae.
- CKH-UBO: A longer incision of approximately 2 cm was made at the desired height determined by C-arm fluoroscopy. After subcutaneous and soft tissue dissection, a non-expanding fixed channel with an inner diameter of 20 mm and an outer diameter of 24 mm (Fig. 1) was placed. C-arm fluoroscopy was used to confirm the appropriate positioning of the fixed channel above the lamina. The residual soft tissue on the bone surface was dissected using an electric knife to identify the lamina and lateral bony landmarks. Under direct visualization, the field within the retractor was cleared, ultimately exposing the V-point.

After exposing the V-point, the two procedures have similar operative characteristics except for the different methods of decompression using a drill or UBO. Both procedures involve removing the inferior border of the upper lamina, the intersection of the upper and lower laminae, and the superior border of the lower lamina, at the V-point, until the attachment of the ligamentum flavum is exposed. Subsequently, the ligamentum flavum and interlaminar ligament are excised to expose the exiting nerve root and the intervertebral disc space below. The medial wall of the articular process is palpated using a nerve hook to avoid excessive resection of the facet joint. Once the freely exposed nerve root is explored in the axilla, shoulder, ventral aspect, and intervertebral disc (if there is protrusion, it is removed using a pituitary rongeur), the inner margin of the spinal cord is observed to ensure sufficient decompression before gently retracting the nerve root.

Successful decompression of the nerve root canal is indicated by a feeling of freedom when palpating the retracted nerve root with a nerve hook. Finally, both procedures achieve complete haemostasis after thorough exploration and decompression of the nerve. The working channels are removed, and layered closure is performed with the placement of a drainage tube. On the first postoperative day, the drainage tube is removed when the draining fluid is less than 50 ml. On the second day postoperatively, cervical X-rays and three-dimensional CT scans are performed for follow-up. The patients can sit up with the assistance of a cervical collar if these imaging examinations are satisfactory. If there are no abnormal findings at the incision site, the patient can be discharged four to five days after surgery. After 1 month, the cervical collar can be removed, and normal neck movement can be resumed.

### Outcome measurements

The clinical data were obtained through database review and questionnaires collected during regular follow-up outpatient visits. A Visual Analog Scale (VAS) for neck and arm, as well as the Neck Disability Index (NDI), was used to evaluate clinical outcomes. At the final follow-up, the overall results were categorized according to the modified Macnab criteria by independent investigators [17] into excellent (no pain, no activity limitation), good (occasional radicular pain, symptomatic relief), fair (improvement in functional ability, but still disabled), and poor (insufficient improvement, requiring further surgical intervention).

Perioperative data analysis included surgical duration, length of hospital stay, intraoperative blood loss, surgical incision length, postoperative complications, and cases requiring reoperation, which were recorded.

**Fig. 2:**
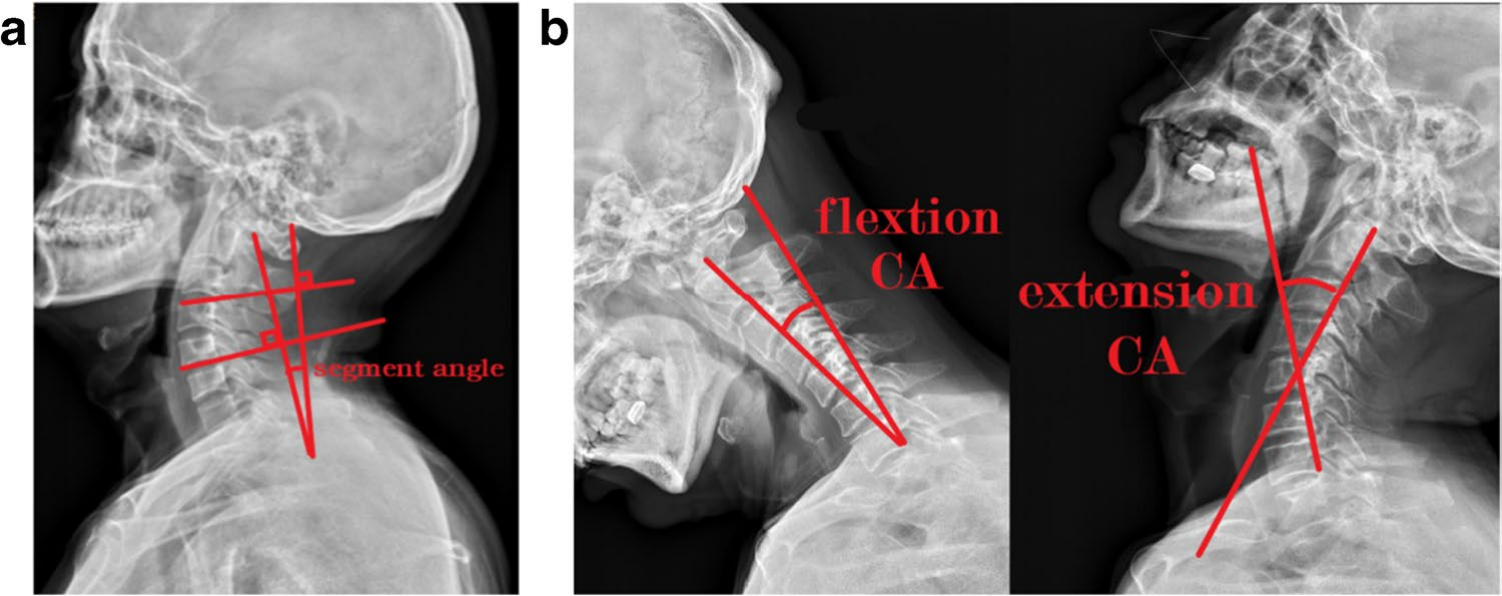
**a** The segmental angle is defined as the angle between the superior border of the upper vertebral body and the inferior border of the lower vertebral body, extended as lines. **b** Range of motion represents the difference in cervical angles (CA) between the hyperextended and hyperflexed positions of the cervical spine (angle between the posterior borders of C2 and C7 extended as lines)

Radiological evaluation primarily focused on stability assessment, including preoperative, three month postoperative, and 1-year postoperative segmental angle (SA) and range of motion (ROM) comparisons, the measurement methods of which are illustrated in Fig. 2.

### Statistical analysis

The data analysis was performed using SPSS 21.0 (SPSS Inc., Chicago, IL, USA) statistical software. The measurement data were presented as mean ± standard deviation. Both groups of patients had data that followed a normal distribution. A two-sample *t*-test was employed to compare the differences in efficacy indicators (arm pain VAS, neck pain VAS, NDI) between the two groups at different time points. A chi-square test for continuous data was used to analyze the differences in gender, BMI, and affected side between the two groups. The Person chi-square test was used to analyze the results of the modified Macnab criteria in the two groups. A *p*-value less than 0.05 was considered statistically significant.

## Results

**Fig. 3:**
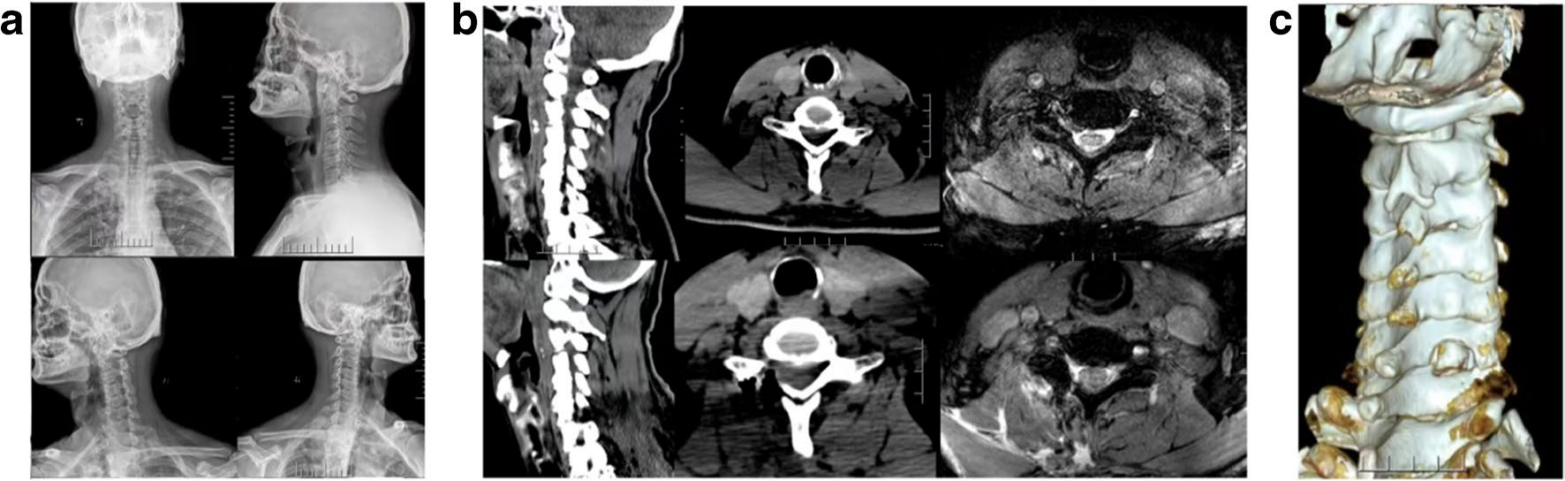
A 47-year-old male patient with right-sided neck pain accompanied by numbness in the upper limb. The surgical segment involved was C6/7. Following surgery, the patient experienced complete alleviation of symptoms. **a** The patient’s cervical spine X-ray in both the lateral and oblique views. **b** A comparison of the patient’s preoperative and postoperative CT and MRI images, with the upper portion representing the preoperative images and the lower portion representing the postoperative images. **c** A postoperative three-dimensional reconstruction of the patient’s cervical spine CT scan

The clinical characteristics of patients in two groups were shown in Table 1. As shown in Table 2, the neck and hand pain scores in the CKH-UBO group improved from 5.86 ± 1.0 and 6.22 ± 0.9 preoperatively to 0.6 ± 0.4 and 0.6 ± 0.5 at one year postoperatively. In the PPECD group, the scores improved from 5.7 ± 1.0 and 6.0 ± 1.0 preoperatively to 0.5 ± 0.4 and 0.7 ± 0.4 at one year postoperatively. The NDI scores in both groups improved from 35.8 ± 3.32 (CKH-UBO group) and 36.6 ± 3.3 (PPECF group) preoperatively to 1.7 ± 1.3 and 2.1 ± 1.3 at one year postoperatively. According to the improved Macnab efficacy evaluation criteria, the clinical success rate was 97.14% in the CKH-UBO group (excellent: 24 cases, good: 10 cases, fair: 1 case, poor: 0 cases) and 92.5% in the PPECF group (excellent: 28 cases, good: 9 cases, fair: 2 cases, poor: 1 case). There was no statistically significant difference between the two groups (*p* = 1, > 0.05). In the CKH-UBO group, one patient experienced a complication similar to nerve injury, which completely recovered within six months. In the PPECF group, one patient had a recurrence at six months postoperatively, and in the same group, two other patients did not experience complete resolution of neck pain and numbness within six months. Figure 3 shows a  typical case of the CKH-UBO group,to represent the imaging comparison before and after the surgery, similar imaging findings were observed in PPECF group.

**Table 1:** The baseline data comparison between the two groups. The age ranges of 35 patients in the CKH-UBO group and 40 patients in the PPECD group were 29–76 years and 25–79 years, respectively. There was no difference in baseline data between the two groups

	CKH-UBO	PPECF	Total	*p* value
Age (years)	52.4 ± 14.3	54.2 ± 15.2	53.4 ± 14.7	0.593
Sex (M:F)	22/13	27/13	49/26	0.673
BMI (kg/m^2^)	22.5 ± 2.0	22.3 ± 2.1	22.4 ± 2.1	0.562
Level (*n*)				
C 3–4	2	2	4	
C 4–5	3	5	8	
C 5–6	18	20	38	
C 6–7	12	13	25	
Side (R:L)	17/18	19/21	36/39	0.926
Blood loss (ml)	32.6 ± 3.5	30.9 ± 3.8	31.7 ± 3.7	0.065
Hospital stay (days)	5.3 ± 1.3	5.1 ± 1.0	5.2 ± 1.1	0.835

**Table 2:** Clinic outcomes

	CKH-UBO	PPECF	*p* value
Neck pain VAS			
Preoperation	5.8 ± 1.0	5.7 ± 1.0	0.398
3 days	1.7 ± 0.5	1.9 ± 0.7	0.146
3 months	1.3 ± 0.7	1.1 ± 0.7	0.170
1 year	0.6 ± 0.4	0.5 ± 0.4	0.504
Arm pain VAS			
Preoperation	6.2 ± 0.9	6.0 ± 1.0	0.347
3 days	1.5 ± 0.5	1.7 ± 0.6	0.170
3 months	1.2 ± 0.7	1.1 ± 0.6	0.173
1 year	0.6 ± 0.5	0.7 ± 0.4	0.357
NDI			
Preoperation	35.8 ± 3.2	36.6 ± 3.3	0.996
3 days	8.9 ± 4.2	10.6 ± 3.4	0.067
3 months	2.8 ± 1.8	2.3 ± 1.7	0.149
1 year	1.7 ± 1.3	2.1 ± 1.3	0.195

**Fig. 4:**
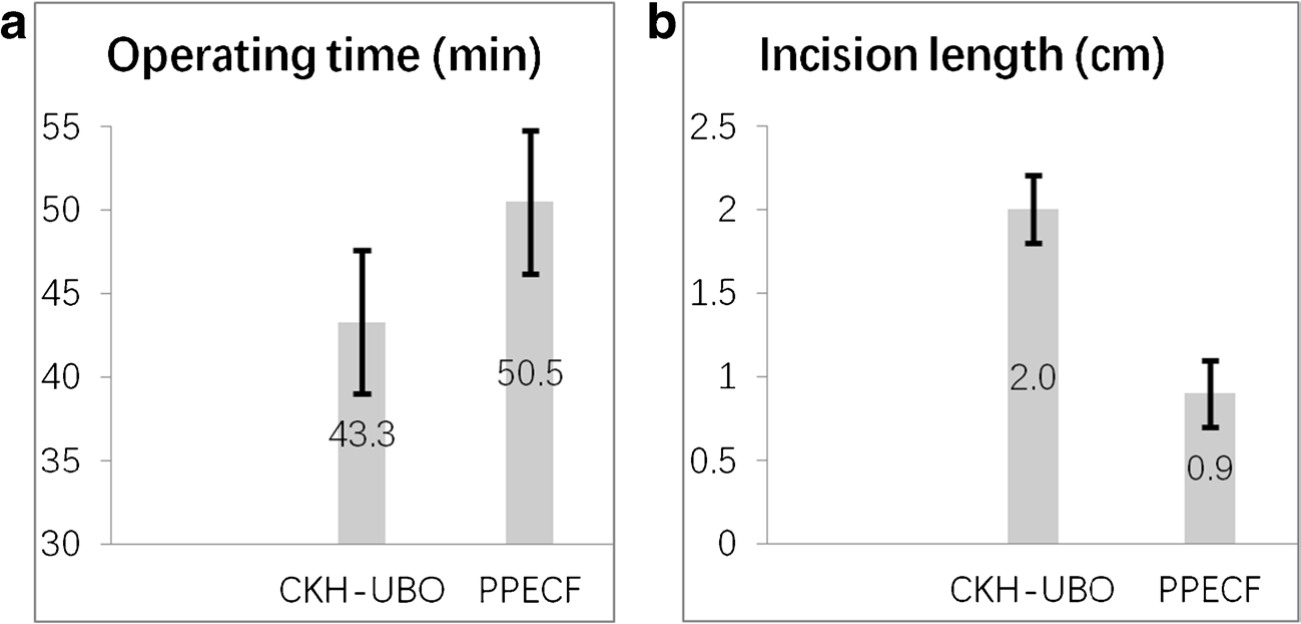
**a** The average operation time for the CKH-UBO group was 43.3 ± 4.3 min, while for the PECF group it was 50.5 ± 6.2 min (*p* = 0.000, < 0.05). **b** Regarding incision length, the CKH-UBO group and the PECF group had lengths of 2.0 ± 0.2 and 0.9 ± 0.2 cm, respectively (*p* = 0.000, < 0.05)

In terms of efficiency, there were no significant differences in the average length of hospital stay and average blood loss (Table 1) between the two groups (*p* > 0.05). However, the CKH-UBO group had significantly shorter surgical duration compared to the PECF group, while the PPECF group had significantly smaller incision length compared to the CKH-UBO group (Fig. 4). In terms of safety and complications, the preoperative, three month, and one year changes in SA (segmental angle) and ROM (range of motion) for each patient did not exceed 2°. There were no significant differences in SA and ROM data at the same stages between the CKH-UBO and PPECF groups (Table 3). Postoperatively, in the CKH-UBO group, one patient experienced new-onset pain and hypersensitivity in the arm. Within six months postoperatively, the symptoms in the pain and hypersensitivity area completely resolved. No other patients experienced complications such as nerve or vascular injury or worsening cervical symptoms in either group.

**Table 3:** SA and ROM comparison

	CKH-UBO	PPECF	*p* value
SA (°)			
Preoperation	4.1 ± 0.3	4.2 ± 0.2	0.078
3 months	4.5 ± 0.1	4.6 ± 0.1	0.094
1 year	4.9 ± 0.1	4.7 ± 0.2	0.169
ROM (°)			
Preoperation	53.3 ± 3.2	52.1 ± 3.2	0.078
3 months	50.4 ± 2.5	50.9 ± 3.1	0.085
1 year	54.2 ± 3.5	51.7 ± 4.3	0.378

## Discussion

The key point of MIS-PCF surgery is to adequately decompress the nerve root canal, which involves thorough removal of the narrow areas around the inner and outer orifices of the nerve root canal, as well as the shoulder, axillary, ventral, and dorsal regions, creating an appropriate space for nerve root movement. Previous studies have shown a close relationship between adequate decompression and the short-term alleviation of preoperative symptoms, as well as the residual symptoms before surgery and the occurrence of long-term recurrence during follow-up [18, 19].

We believe that the effect of visualizing and clearing the shoulder and axillary regions of the nerve root under the guidance of UBO and using a large channel is comparable to that of microscopic techniques. The spoon-shaped blade used in this technique directs the force away from the nerve root when removing osteophytes, greatly reducing the interference with the nerve during surgery and allowing surgeons to clear the osteophytes that may cause stimulating symptoms to the nerve root without any concerns, demonstrating the advantages of decompression.

In terms of safety, there were no major surgical complications observed in either group. From the actual operation comparing UBO and grinding, UBO did offer advantages in avoiding mechanical damage, but for thermal injury, both methods had their pros and cons. In PPECF, the flowing physiological saline at room temperature served as a cooling medium, but the cooling reaction was slow, and the temperature could easily rise again in a short period. On the other hand, CKH-UBO used air as a medium, providing good thermal isolation, and the heat transfer could be immediately interrupted by removing the blade, thus minimizing the threat of thermal injury to the nerve root. However, the disadvantage was that the local temperature might be higher compared to when water is used as a medium.

In one case in our study, the ultrasonic bone scalpel was used locally for an extended period, resulting in local temperature elevation. We immediately removed the scalpel and used physiological saline for cooling, but the patient still experienced hyperalgesia in the upper arm area postoperatively, which we believed might be caused by the local temperature elevation. However, the patient’s wound healing and upper limb blood supply were not significantly affected, and the hyperalgesic area gradually subsided after two months postoperatively.

In this study, we found that the stability of patients was not significantly affected by either surgical approach. Stability is directly related to the preservation of small joints, and posterior approach surgery causes less disruption to stability compared to anterior approaches [20, 21]. Studies have shown that to achieve decompression and maintain stability, resection of at least 25% or more of the medial facet joints is typically necessary, with a minimum of 50% removal recommended [6, 22]. Under microscopic or endoscopic visualization, surgeons have a closer view to observe and ensure the accuracy of bone resection. Similarly, with the assistance of UBO via a larger channel, the accuracy of bone resection can be adequately ensured.

We believe that CKH-UBO has a significant advantage in terms of surgical efficiency. UBO reduces the surgeon’s concerns about nerve injury, significantly reducing the time spent on determining the safe distance for nerve root damage. The combination of the ultrasonic bone scalpel and the large channel maximizes the safety of UBO and eliminates the need for endoscope installation, further enhancing efficiency. Under the large channel, electric surgical knives and tools of the same size as those used in open surgery can be used to quickly remove soft tissues around the facet joints and vertebral plate edge [16], expanding the operative field and workspace. Simultaneously, the surgeon can manipulate the ultrasonic bone scalpel with one hand while using a retractor with the other hand to maintain a clear surgical field of view. In PPECF, to prevent displacement of the grinding head, both hands must operate simultaneously, which increases the difficulty of the surgical procedure and the associated physical fatigue. Furthermore, direct visualization eliminates the need to separate the eyes and hands under a microscope or endoscope, reducing surgical complexity. Most steps are similar to open surgery, which also shortens the learning curve for the procedure [23].

## Conclusion

Based on our comparative results and analysis, we believe that both CKH-UBO and PPECF are advanced, efficient, and safe minimally invasive surgical approaches in today’s spine surgery field. They have similar clinical outcomes and safety profiles, with individual advantages in terms of efficiency. CKH-UBO saves more time but requires patients to undergo larger incisions. From the perspective of individual surgeons, understanding and utilizing the approach that they are most skilled in is the most efficient. The advantages and disadvantages of different surgical approaches may require longer-term follow-up, larger sample sizes, or randomized controlled trials for further validation. As spinal minimally invasive techniques continue to evolve, there are more surgical approaches available. We believe that regardless of the method of surgical improvement, we are constantly advancing towards a goal of high-efficiency and safe procedures.

## Data Availability

The datasets used during the current study are available from the corresponding author on reasonable request.
